# Domestic cooking methods affect nutrient, phytochemicals, and flavor content in mushroom soup

**DOI:** 10.1002/fsn3.996

**Published:** 2019-05-15

**Authors:** Yujing Sun, Feiyan Lv, Jinhu Tian, Xing qian Ye, Jianchu Chen, Peilong Sun

**Affiliations:** ^1^ Department of Food Science and Technology, Ocean College Zhejiang University of Technology Hangzhou China; ^2^ Department of Food Science and Nutrition, School of Biosystems Engineering and Food Science Zhejiang University Hangzhou China

**Keywords:** cooking, mushroom soup, nucleotides, polyphenols, polysaccharides

## Abstract

The effects of different cooking methods, including autoclaving, microwaving, sous vide, and stewing, on the nutritional quality of mushroom (*Hypsizygus marmoreus*) soup were investigated. The results showed that all four cooking methods increased the polysaccharide, polyphenol, and amino acid levels compared to uncooked soup. Stewing increased protein content with the other cooking methods showing no change when compared with uncooked soup. Sous vide increased nucleotide content with the other methods decreasing nucleotide levels, and this method was also the best for increasing polyphenol and flavor compounds. Autoclaving generated the highest levels of polysaccharides. In summary, each method had a characteristic effect on mushroom soup properties, and cooking improved the nutritional value of mushrooms by the increase in releasing macro‐ and micronutrients.

## 1 INTRODUCTION

Soup is a liquid culinary preparation generally composed of boiled water, vegetables, poultry, meat, or seafood. It is a staple diet of billions around the globe and exists in many traditional varieties. Soup is a major part of the Chinese culinary tradition. There are several types of soup, including vegetable, chicken, fish, or mixture of these ingredients. Vegetable soup is recognized by many Chinese people as a popular way of consuming vegetables, which promotes a healthy diet and lifestyle.

Mushroom soup is a traditional vegetable soup in China, which has been consumed since ancient times for its nutritive value, flavor properties, and functional properties. Nutritionally, mushrooms provide key nutrients, amino acids, monosaccharides, dietary fiber, and many bioactive components. Recent reports indicated that edible mushroom extracts exhibit promising therapeutic and health‐promoting benefits, particularly in relation to diseases associated with inflammation (Muszyńska, Grzywacz‐Kisielewska, Kała, & Gdula‐Argasińska, 2018), anticancer activities, anti‐atherosclerotic, antihypertensive, and cholesterol lowering effects, anti‐aging and antioxidant properties (Ren, Perera, & Hemar, 2012; Schneider et al., 2011; Tang et al., 2016). With respect to organoleptic properties, Tsai, Tsai, and Mau (2008) reported that the taste of a mushroom extract was primarily attributed to free amino acids, nucleotides, and soluble sugars.

However, most research has been conducted on a mushroom extract with little investigation centered on cuisine‐generated mushroom soup. To the best of our knowledge, studies on bioactives, phytochemicals, and nutrients in mushroom soup are limited. Li et al. (2011) reported the flavor of mushroom soup generated from button mushroom powder was dependent on the cooking method, with the levels of free amino acids and 5′‐nucleotides in microwaved mushroom soup higher than those in boiled or autoclaved soup. The number of volatile compounds identified in boiled mushroom soup was higher than those in autoclaved and microwaved soup. Tan et al. (2015) reported that the enhancement or reduction of polyphenols and antioxidant activities of oyster mushroom soup was attributed to the cooking method and mushroom variety. Sun, Bai, and Zhuang (2014) determined that boiling significantly decreased total phenolics and improved the antioxidant activities in four *Boletus* sp.

The objective of this study was to analyze the influence of traditional and modern domestic cooking methods on nutrition, flavors, and bioactive compounds in mushroom (*Hypsizygus marmoreus*) soup.

## 2 MATERIALS AND METHODS

### 2.1 Materials and soup preparation

Fresh mushroom (*H. marmoreus*) was obtained from a local supermarket (Hangzhou, China). Mushrooms were washed, drained, had the stipe end removed, and then randomly assorted into 200 g samples for cooking, and the samples were added into various cookers with water in a proportion of one part mushroom to six parts water for autoclaving, microwaving, sous vide, and stewing. For the microwave method, the mushrooms were cooked in a commercial 780 W microwave (W25800K‐01AG; Fotile, China) for 8 min. For stewing, mushrooms were first brought to boil using an electromagnetic oven (H18S012; Midea, China) and then boiled using a water bath (HH‐10; Kejie, China) at 90°C for 90 min. Pressure‐cooked mushrooms were autoclaved using pressure cooker (D22E1; Supor, China) for 20 min at 120°C. For sous vide, mushrooms were added into a retort pouch and vacuumed and then boiled at 60°C using a water bath (HH‐10; Kejie) for 4 hr. After cooking, all the samples including the cooking water were cooled to room temperature (25 ± 2°C), and cooked mushrooms were drained, then freeze‐dried (FreeZone 6; Labconco, USA), ground using a commercial grinder (Wenling Linda Machine Co., China), sealed with aluminum foil, and stored at −18°C for further analysis. Water derived from cooking was concentrated using an evaporimeter and stored at −18°C for further analysis.

### 2.2 Determination of total polysaccharide content and crude protein

Polysaccharide content was determined by the phenol‐sulfuric acid method using glucose as standard (NY/T 1676‐2008; Determination method of crude polysaccharide in edible fungus). Freeze‐dried mushroom powder (0.500 g) was added to a 50‐ml centrifuge tube, then 5 ml of water was added, and 20 ml of 100% ethanol was added slowly. The sample was blended using a vortex oscillator, extracted for 30 min by ultrasound, and centrifuged for 10 min at 3,800 g. The supernatant was discarded and the precipitate washed and resuspended in 80% ethanol and transferred to a round bottom flask, to which 50 ml of distilled water was added. An air condenser was inserted into the round bottom flask and the material was extracted for 2 hr in a boiling water bath, then cooled to 25°C, and filtered. The supernatant was transferred to a 250‐ml volumetric flask, and the insoluble material was washed three times with water. A final volume of 250 ml of washed extract was obtained, which was termed as the mushroom extract. A volume of 5 ml of concentrated soup was added to a 50‐ml centrifuge tube to which 20 ml of 100% ethanol was slowly added. The sample was blended using a vortex oscillator and then centrifuged for 10 min at 4,000 rpm. The supernatant was discarded, and the insoluble material was washed and centrifuged in 80% (*v*/*v*) ethanol.

For carbohydrate determination, 0.5 ml of mushroom extractions and mushroom soup extractions was separately added to 20‐ml tubes, and then, 0.5 ml of distilled water was added, followed by 0.1 ml of 5% (*v*/*v*) phenol solution and 5.0 ml H_2_SO4 and then incubated for 10 min. The sample was then blended using a vortex oscillator and incubated for 20 min in a 30°C water bath, and carbohydrate levels were determined using a spectrophotometer at 490 nm using glucose as a standard.

The protein content was determined using the Kjeldahl method (GB/T 5009.5‐2003; Determination method of food protein.). Freeze‐dried mushroom powder (0.200 g) or 1 ml of concentrated soup was added to a digestive tube separately, and then, 0.200 g CuSO_4_, 3.000 g K_2_SO_4_, and 10 ml H_2_SO_4_ were added to the two tubes. The tubes were capped, shaken, and heated on a digestion furnace for 40 min at 120°C, then for 40 min at 240°C, and then for 1 hr at 360°C. Finally, the temperature was adjusted to 420°C for 30 min when the liquid in the digestive tube was typically bluish‐green and transparent. The tubes were then cooled, and the protein was determined using a Foss‐2300 nitrogen analyzer.

### 2.3 Total phenolic content analysis

Freeze‐dried mushroom powder (2.000 g) was extracted twice with 20 ml of 60% (*v*/*v*) ethanol in an ultrasound bath (150 W) at 25°C for 20 min. After centrifugation at 4,400 g for 15 min, the supernatants were combined and adjusted to 20 ml for measurement of the total phenolic content (TPC).

The TPC was determined by the Folin–Ciocalteu method (Sommer, Schwartz, Solar, & Sontag, 2009). Briefly, 0.5 ml mushroom extraction and 0.5 ml mushroom soup separately were added to a 25‐ml colorimetric cylinder containing 10 ml water and 0.5 ml Folin–Ciocalteu reagent and then mixed well. After 5 min, 5 ml of 5% Na_2_CO_3_ solution was added and mixed with a vortex shaker, using distilled water to adjust the total volume to 25 ml. After 60 min, the absorbance at 750 nm was measured in a UV‐2550 spectrophotometer (Shimadzu Co, Kyoto, Japan) using distilled water as a blank. A calibration curve was prepared by using a standard solution of gallic acid, and the results of total phenols were expressed as mg gallic acid equivalents (GAE) per 100 ml of juice.

### 2.4 Free amino acid analysis

The amino acid composition was analyzed according to the report of Norziah and Ching (2000), using the Waters Associates AccQ‐Tag method. 1.000 g freeze‐dried mushroom powder was diluted with 20 ml of deionized water, and then, 20 ml trichloroacetic acid (5%, *v*/*v*) was added and incubated at 4°C for 12 hr. This was then filtered and adjusted to pH 6 in a 50 ml volume. 5 ml of this sample as described above was filtered through 0.45‐μm polyvinylidene fluoride microfiltration membrane (Shanghai Xingya Purification Material Co., Shanghai, China) and precolumn derivatized with the AccQfluor reagent. The mushroom soup was centrifuged at 4,900 g for 10 min, and the collected supernatant liquid was filtered through a 0.45‐μm polyvinylidene fluoride microfiltration membrane and derivatized as described above. All the derivatized samples were analyzed by Nova‐Pak TMC18 (150 × 3.9 mm, 4 μm). The mobile phase was the AccQ‐Tag Eluent (10%, *v*/*v*), acetonitrile (100%, *v*/*v*), and ultrapure water (100%). The injection volume was 10 μL, and the analyte was monitored at 248 nm using a Waters e2695 Separations Module equipped with a Waters 2489 UV/Vis Detector.

### 2.5 Nucleotide assay

Nucleotides were extracted using a modified method of Liu et al. (2014). Freeze‐dried mushroom powder (1.000 g) was extracted with 20 ml of distilled water and heated to the boiling temperature for 1 min. Then, it was cooled to room temperature, centrifuged at 9,850 g for 15 min and then filtered with a 0.45‐μm polyvinylidene fluoride microfiltration membrane (Shanghai Xingya Purification Material Co.) for HPLC analysis. The mushroom soup was centrifuged at 5,000 rpm for 10 min, and the liquid supernatant was filtered as described above. The nucleotide was analyzed using a Waters Atlantis C_18_ column (250 × 4.6 mm, 5 μm). The mobile phase was 0.01 M KH_2_P0_4_ buffer solution including 1.40 mM tetra‐n‐butylammonium hydrogen sulfate (A) and methanol (B), and the flow rate was 1 ml/min. All samples were detected at 254 nm using a Waters e2695 Separations Module equipped with a Waters 2489 UV/Vis Detector.

### 2.6 Microstructure of cooked and fresh mushroom

A scanning electron microscope (*SEM*) was used to examine the changes in the physical structure of cooked mushroom. Briefly, the samples were collected after cooking, separated into mushroom cap and stipe, immediately soaked in liquid nitrogen, and freeze‐dried using a freeze‐dryer (FreeZone 6; Labconco). The freeze‐dried samples were mounted on the stub, sputter coated with gold in a sputter coater (SCD 050; Balzers, Liechtenstein), and examined with a microscope (TM‐1000; Hitachi, Tokyo, Japan).

### 2.7 Statistical analysis

All samples were prepared and analyzed in triplicate, and the results are presented as the mean ± *SD*. Analysis of variance was used to determine statistical significance. Significant difference between means was determined by least significant difference. Pearson's correlation coefficient (*R*) and *p*‐value were used to show correlations and their significance. A value of *p* < 0.05 was assumed to be a statistically significantly different (SPSS for Windows, version 15.0; Chicago, IL, USA).

## 3 RESULTS AND DISCUSSION

### 3.1 Effect of different cooking methods on protein levels

**Figure 1:**
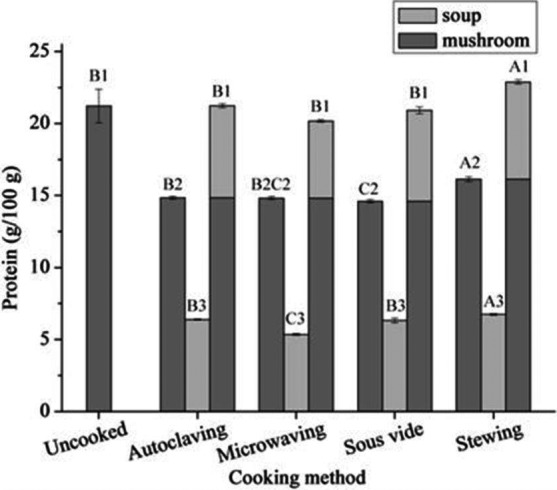
The contents of proteins of mushroom and soup at four cooking methods

The influence of cooking methods on the protein level of mushroom soup is shown in Figure 1. Stewing significantly improved the levels of total protein (22.89 g/100 g DW) in mushrooms and soup (*p* ≤ 0.05), and autoclaving, microwaving, and sous vide did not decrease the total protein compared to the fresh mushrooms (21.22 g/100 g DW). The highest content of protein from mushrooms or mushroom soup was obtained using stewing, and the lowest content of protein from mushroom and mushroom soup was obtained from sous vide and microwaving, respectively. In general, the levels of protein in mushrooms were higher than those detected in soup using any cooking method employed in the present study.

### 3.2 Effect of different cooking methods on polysaccharide levels

**Figure 2:**
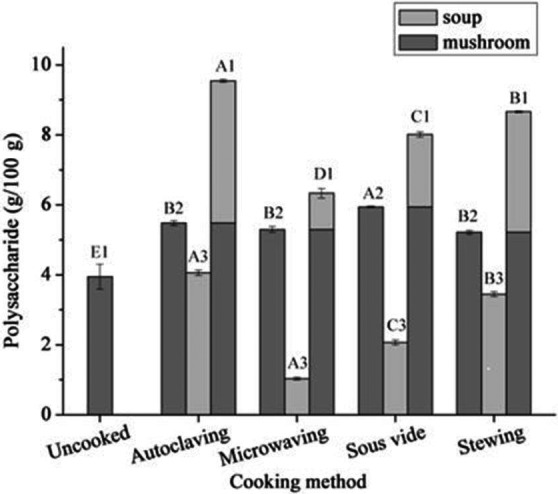
The contents of polysaccharides of mushroom and soup at four cooking methods

Figure 2 shows the variability in polysaccharide content when using different cooking methods. Total polysaccharides in mushroom and soup obtained after any of the four cooking methods were all significantly higher than uncooked mushrooms and were significantly different among the four cooking methods (*p* < 0.05). The mushroom and soup after autoclaving had the highest total polysaccharide content (9.54 g/100 g DW), and the mushrooms and mushroom soup when microwaved had the lowest total polysaccharides (6.33 g/100 g DW). The highest level of polysaccharides in mushrooms was after sous vide cooking, and the levels detected after cooking with the other three methods were similar. The highest polysaccharide level in soup was after autoclaving (4.06 g/100 g DW) which was similar to uncooked mushrooms while the lowest levels were detected after microwaving (1.035 g/100 g DW). The difference in polysaccharide levels can be explained as microwaving has a minimal level on cell destruction, while autoclaving operated under high temperature and pressure causes significant cell and subcellular damage. Unsurprisingly, the content of polysaccharides in mushrooms was higher than in soup after any cooking method used in the present study.

### 3.3 Effect of different cooking methods on total phenolics

**Figure 3:**
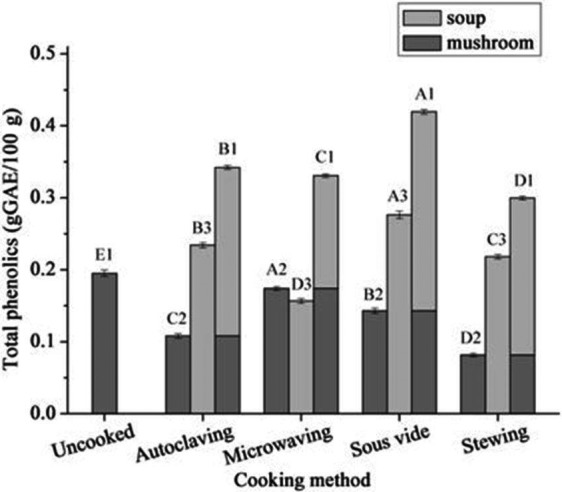
The contents of total phenolics of mushroom and soup at four cooking methods

The differences in phenolic content of mushrooms and soup among the four cooking methods are summarized in Figure 3. It was determined that the four cooking methods all improved levels of total phenolics in mushrooms and mushroom soup (*p* ≤ 0.05) compared to uncooked mushrooms. The highest phenolic levels were in mushrooms and soup after sous vide, and the lowest after stewing. The mushroom sample had the highest phenolics after microwaving and had the lowest with stewing. Mushroom soup had the highest phenolics after sous vide cooking and the lowest with microwaving. The content of phenolics in soup was higher than that in mushroom using the four cooking methods except microwaving. It has been established that polyphenols are sensitive to heat and pressure (Azizah, Wee, Osman, & Misran, 2009; Zhang & Hamauzu, 2004) so the results obtained here could be influenced by cooking methods. For example, the mushrooms after sous vide cooking had higher dissolution and retention compared with others cooking methods and this may be related to lower cooking temperatures and oxygen concentrations. The results for the microwave method may be due to minimal cell destruction and a short treatment time, which does not destroy the molecules in the present investigation.

### 3.4 Effect of cooking methods on free amino acid levels

Table 1 displays the free amino acids (FAAs) of mushrooms and mushroom soup after different cooking methods. The total FAA content of mushroom and mushroom soup in the four cooking methods was significantly higher than fresh mushroom (*p *<* *0.05), and the total content was highest using sous vide and autoclaving, and the lowest was the microwave method. Analysis of the FAA levels in soup revealed a value of (1.837 mg/g DW) after autoclaving, which was significantly higher than the value determined after sous vide (0.901 mg/g DW), stewing (0.869 mg/g DW), and microwaving (0.371 mg/g DW). The total FAA in mushrooms was higher than in mushroom soup after any cooking method used in the present study. Tyr and Lys were not detected in the mushrooms and mushroom soup, but there were many flavor amino acids, including the umami amino acids, Glu, Asp, and sweet tasting amino acids, including Thr, Ser, Pro, Gly, and Ala, which were the highest in mushroom soup after autoclaving and in mushrooms and mushroom soup after sous vide cooking. Free amino acids in soup may have originated from the physical degradation of mushrooms and the hydrolysis of protein.

**Table 1:** Effect of different cooking methods on the free amino acids (FAAs) of mushroom and soup

FAA (mg/g)	Uncooked	Autoclaving		Microwaving		Sous vide		Stewing	
	Mushroom	Mushroom	Soup	Mushroom	Soup	Mushroom	Soup	Mushroom	Soup
Asp	0.398 ± 0.033	0.378 ± 0.005^d^	0.279 ± 0.006^B^	0.776 ± 0.005^a^	0.090 ± 0.006^D^	0.898 ± 0.004^c^	0.188 ± 0.003^C^	1.344 ± 0.008^b^	0.298 ± 0.003^A^
Ser	0.143 ± 0.015	0.266 ± 0.004^d^	0.089 ± 0.003^B^	0.379 ± 0.004^b^	0.040 ± 0.003^D^	0.566 ± 0.003^a^	0.111 ± 0.004^A^	0.385 ± 0.005^c^	0.081 ± 0.004^C^
Glu	0.290 ± 0.025	0.409 ± 0.003^d^	0.190 ± 0.002^C^	0.676 ± 0.005^c^	0.100 ± 0.006^D^	1.108 ± 0.004^a^	0.244 ± 0.005^A^	0.902 ± 0.006^b^	0.204 ± 0.003^B^
Gly	0.084 ± 0.009	0.106 ± 0.004^d^	0.043 ± 0.002^B^	0.154 ± 0.004^c^	0.018 ± 0.004^A^	0.245 ± 0.004^a^	0.045 ± 0.004^B^	0.204 ± 0.008^b^	0.037 ± 0.004^B^
His	0.276 ± 0.029	0.970 ± 0.004^c^	0.827 ± 0.004^A^	0.886 ± 0.005^d^	0.195 ± 0.006^D^	2.169 ± 0.006^a^	0.429 ± 0.008^B^	1.388 ± 0.005^b^	0.283 ± 0.005^C^
Arg	0.281 ± 0.023	1.145 ± 0.006^d^	1.613 ± 0.009^A^	1.578 ± 0.007^b^	0.157 ± 0.007^D^	1.885 ± 0.007^a^	0.373 ± 0.008^B^	1.522 ± 0.009^c^	0.316 ± 0.007^C^
Thr	0.121 ± 0.010	0.256 ± 0.003^c^	0.298 ± 0.006^A^	0.287 ± 0.009^b^	0.033 ± 0.006^D^	0.429 ± 0.007^a^	0.080 ± 0.003^B^	0.296 ± 0.004^b^	0.056 ± 0.004^C^
Ala	0.236 ± 0.017	0.326 ± 0.004^d^	0.560 ± 0.007^A^	0.452 ± 0.007^c^	0.056 ± 0.005^D^	0.776 ± 0.003^a^	0.162 ± 0.004^B^	0.546 ± 0.007^b^	0.119 ± 0.005^C^
Pro	0.155 ± 0.012	0.168 ± 0.003^d^	0.379 ± 0.004^A^	0.290 ± 0.005^c^	0.035 ± 0.005^C^	0.403 ± 0.008^a^	0.073 ± 0.003^B^	0.381 ± 0.005^b^	0.073 ± 0.004^B^
Cys‐s	0.119 ± 0.007	0.170 ± 0.004^c^	0.165 ± 0.006^A^	0.181 ± 0.006^b^	0.013 ± 0.003^D^	0.359 ± 0.003^a^	0.065 ± 0.002^B^	0.141 ± 0.003^d^	0.027 ± 0.003^C^
Tyr	ND	ND	ND	ND	ND	ND	ND	ND	ND
Val	0.096 ± 0.008	0.133 ± 0.006^b^	0.129 ± 0.006^A^	0.109 ± 0.004^c^	0.010 ± 0.005^D^	0.268 ± 0.003^a^	0.054 ± 0.002^B^	0.107 ± 0.003^c^	0.022 ± 0.002^C^
Met	0.186 ± 0.015	0.859 ± 0.007^b^	0.790 ± 0.008^A^	0.814 ± 0.005^c^	0.079 ± 0.006^D^	1.386 ± 0.011^a^	0.302 ± 0.006^B^	0.776 ± 0.003^d^	0.179 ± 0.003^C^
Lys	ND	ND	ND	ND	ND	ND	ND	ND	ND
Ile	0.074 ± 0.005	0.109 ± 0.003^b^	0.108 ± 0.004^A^	0.100 ± 0.006^c^	0.010 ± 0.001^D^	0.218 ± 0.003^a^	0.043 ± 0.002^B^	0.090 ± 0.004^d^	0.018 ± 0.004^C^
Leu	0.147 ± 0.011	0.229 ± 0.005^b^	0.168 ± 0.003^A^	0.148 ± 0.004^c^	0.016 ± 0.004^D^	0.457 ± 0.004^a^	0.088 ± 0.003^B^	0.141 ± 0.003^d^	0.027 ± 0.004^C^
Phe	0.115 ± 0.011	0.188 ± 0.003^b^	0.181 ± 0.003^A^	0.174 ± 0.007^c^	0.016 ± 0.002^D^	0.363 ± 0.007^a^	0.067 ± 0.002^B^	0.152 ± 0.005^d^	0.030 ± 0.002^C^
Total	2.722 ± 0.229	5.708 ± 0.052^d^	5.818 ± 0.074^A^	7.003 ± 0.079^c^	0.867 ± 0.067^D^	11.531 ± 0.066^a^	2.322 ± 0.053^B^	8.373 ± 0.070^b^	1.771 ± 0.054^C^
Flavor AAs	1.427 ± 0.121	1.920 ± 0.021^d^	1.837 ± 0.030^A^	3.013 ± 0.040^c^	0.371 ± 0.037^C^	4.425 ± 0.032^a^	0.901 ± 0.025^B^	4.056 ± 0.040^b^	0.869 ± 0.027^B^

Notes

ND: not detected.

a–d: significant difference (*p* < 0.05) in different mushroom samples within a row; A–D: significant difference (*p* < 0.05) in different soup samples within a row; flavor AAs: umami AAs (Asp, Glu) + sweet AAs ( Ser，Gly，Thr，Ala，Pro). Results were expressed as means ± *SD* (*n* = 3). Means with different letters within a row were significantly different (*p* < 0.05). ANOVA and Duncan test were used to analyze the significant difference between samples from different cooking methods.

### 3.5 Effect of cooking methods on the nucleotide levels

Nucleotide levels in mushrooms and mushroom soup after different cooking methods are listed in Table 2. The total content of nucleotides (2.193 mg/g DW) in mushrooms and mushroom soup using sous vide was higher than fresh mushroom, and stewing (0.212 mg/g DW), autoclaving (0.309 mg/g DW), and microwaving (0.775 mg/g DW) destroyed the nucleotides in mushroom and mushroom soup. The 5′‐GMP, which generates a meat flavor (Tsai et al., 2008), and 5′‐AMP, which has a sweet flavor (Gu et al., 2011), were detected in the mushrooms and mushroom soup and created a sweet taste of the mushroom soup. It was also determined that the two flavor‐generating nucleotides were highest using sous vide than in other cooking methods. This may be explained by several factors. First, sous vide has a gentle conditions that do not damage nucleotide functionality, and second, 5′‐XMP deaminase retains enzyme activity after sous vide cooking, which ensures 5′‐XMP can still be bioconverted to 5′‐GMP (Schwartz & Margalith, 1973).

**Table 2:** Effect of different cooking methods on the 5′‐nucleotides of mushroom and soup

5′‐nucleotides (mg/g)	Uncooked	Autoclaving		Microwaving		Sous vide		Stewing	
	Mushroom	Mushroom	Soup	Mushroom	Soup	Mushroom	Soup	Mushroom	Soup
5′‐CMP	1.016 ± 0.011	0.248 ± 0^c^	0.127 ± 0.002^B^	0.499 ± 0.001^b^	ND	1.563 ± 0.004^a^	0.734 ± 0.006^A^	0.199 ± 0.001^d^	0.075 ± 0.002^C^
5′‐UMP	0.057 ± 0.003	0.046 ± 0.001^b^	0.026 ± 0.001^B^	0.104 ± 0.003^a^	0.019 ± 0.001^C^	0.205 ± 0.001^d^	0.084 ± 0^A^	0.032 ± 0^c^	0.020 ± 0.001^C^
5′‐IMP	ND	ND	ND	ND	ND	ND	ND	ND	ND
5′‐GMP	0.016 ± 0.001	0.008 ± 0^c^	0.006 ± 0^B^	0.009 ± 0^b^	0.005 ± 0^C^	0.036 ± 0^a^	0.015 ± 0^A^	0.005 ± 0^d^	0.005 ± 0^C^
5′‐XMP	0.064 ± 0.001	0.030 ± 0.002^b^	0.035 ± 0.004^A^	0.072 ± 0^a^	0.010 ± 0^D^	0.030 ± 0^b^	0.016 ± 0^C^	0.026 ± 0^c^	0.020 ± 0^B^
5′‐AMP	0.053 ± 0.004	0.064 ± 0.001^c^	0.040 ± 0.002^B^	0.091 ± 0.001^b^	0.021 ± 0.003^C^	0.359 ± 0.002^a^	0.155 ± 0.001^A^	0.029 ± 0^d^	0.020 ± 0^C^
Total	1.205 ± 0.012	0.309 ± 0.001^c^	0.234 ± 0.005^B^	0.775 ± 0.001^b^	0.060 ± 0^D^	2.193 ± 0.005^a^	1.003 ± 0.008^A^	0.212 ± 0.001^d^	0.140 ± 0.003^C^

Notes

5′‐AMP: 5′‐adenosine monophosphate; 5′‐CMP: 5′‐cytidine monophosphate; 5′‐GMP: 5′‐guanosine monophosphate; 5′‐IMP: 5′‐inosine monophosphate; 5′‐UMP: 5′‐uridine monophosphate; 5′‐XMP: 5′‐xanthosine monophosphate; ND: not detected.

a–d: significant difference (*p* < 0.05) in different mushroom samples within a row; A–D: significant difference (*p* < 0.05) in different soup samples within a row; results were expressed as means ± *SD* (*n* = 3). Means with different letters within a row were significantly different (*p* < 0.05). ANOVA and Duncan test were used to analyze the significant difference between samples from different cooking methods.

### 3.6 The microstructure of mushrooms after different cooking methods

**Figure 4:**
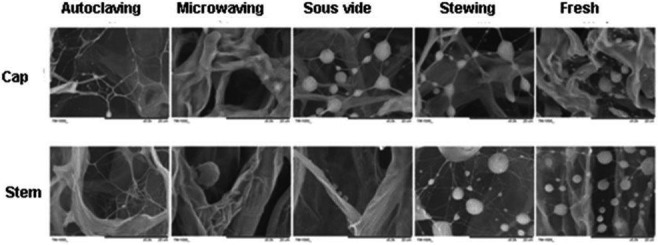
Scanning electron microscopy (5,000×) image of mushroom caps’ and stems’ longitudinal section from different cooking methods

To examine the reasons for variation in the levels of mushroom constituents under different cooking methods, we studied the microstructure of fresh and cooked mushrooms by scanning electron microscopy (*SEM*). The *SEM* images (Figure 4) revealed that fresh mushroom cap and stems appeared as compacted microstructures with many spherical spores and thin mycelial structures. After cooking, the structure of the mushroom caps and stems changed significantly. After autoclaving, due to the high pressure, the spores and mycelium of the mushrooms disappeared, more than that with the three other cooking methods. In microwaving, the spores and mycelium of mushroom also disappeared, and a big cavity appeared, which may have resulted from the selective regional heating of the microwave. However, for stewing and sous vide, the spores and mycelium of mushroom remained largely intact. Overall, the order of subcellular damage and effects on macronutrients and flavor went from autoclaving, microwaving, and stewing to the least damaging method of sous vide cooking.

## 4 CONCLUSIONS

This study showed that all four cooking methods improved the polysaccharide, polyphenol, and amino acid content of mushrooms compared to the uncooked mushrooms. Stewing increased the proteins content. Sous vide significantly increased the nucleotide content. Autoclaving was the best method to generate high levels of polysaccharides. Overall, these findings suggested that each method had a characteristic effect on mushroom soup properties.

## CONFLICT OF INTEREST

The authors declare that they do not have any conflict of interest.

## ETHICAL APPROVAL

This study does not involve any human or animal testing.

## INFORMED CONSENT

Written informed consent was obtained from all study participants.
